# Serum Cytokine Profiles and Their Association with Clinical Severity in Dogs with Atopic Dermatitis

**DOI:** 10.3390/ani16081182

**Published:** 2026-04-13

**Authors:** Jae-Yun Ko, Min-Hee Kang, Hee-Myung Park

**Affiliations:** 1 Pyeonanhan Animal Hospital, Daejeon 34069, Republic of Korea; musoi1l@naver.com; 2 Department of Veterinary Internal Medicine, College of Veterinary Medicine, Konkuk University, Seoul 05029, Republic of Korea; 3 Department of Bio-Animal Health, Jangan University, Hwaseong 18331, Republic of Korea; mhkang@jangan.ac.kr

**Keywords:** canine atopic dermatitis, cytokine, pVAS, CADESI-04, biomarker

## Abstract

Canine atopic dermatitis is a common chronic inflammatory skin disease characterized by pruritus and recurrent erythema, yet objective blood biomarkers for monitoring disease activity remain limited. In this study, we evaluated serum cytokine profiles and their associations with clinical severity in client-owned dogs with atopic dermatitis. A total of 143 dogs were enrolled, including 28 healthy and 115 dogs with atopic dermatitis. The atopic dermatitis group was further categorized into untreated dogs (*n* = 27; no systemic therapy for ≥4 weeks) and systemically treated dogs (*n* = 88). Serum concentrations of IFN-γ, IL-10, IL-13, IL-31, and TGF-β1 were measured using an enzyme-linked immunosorbent assay. Group differences were assessed using the Kruskal–Wallis test with Bonferroni-adjusted post hoc comparisons, and correlations with the pruritus visual analog scale (pVAS) and the Canine Atopic Dermatitis Extent and Severity Index-04 (CADESI-04) were analyzed using Spearman’s rank correlation. Serum IFN-γ, IL-13, and IL-31 concentrations differed significantly among groups (*p* < 0.001, *p* = 0.001, and *p* = 0.004, respectively). IFN-γ and IL-13 concentrations were lower in treated dogs than in healthy dogs and untreated dogs, whereas IL-31 concentrations were higher in dogs with atopic dermatitis than in healthy dogs, regardless of treatment status. In correlation analyses, the pVAS showed a negative correlation with IFN-γ (r = −0.239, *p* = 0.004) and a positive correlation with IL-31 (r = 0.173, *p* = 0.039), while CADESI-04 showed a negative correlation with IFN-γ (r = −0.252, *p* = 0.002). IL-10 and TGF-β1 did not show significant differences among groups or correlations with clinical indices. These findings suggest that serum IL-31 may reflect pruritus-related immune signaling that can persist despite clinical improvement. While IFN-γ may show a weak negative correlation with clinical severity indices, its potential association with chronic dermatologic changes, such as lichenification, requires further investigation in relation to disease chronicity. Together, these results indicate that circulating cytokine profiles and clinical indices do not necessarily change in parallel and that selected cytokines may provide complementary information when interpreting disease activity in canine atopic dermatitis. These profiles should be interpreted while considering the diverse immunomodulatory mechanisms of the systemic therapy administered.

## Simple Summary

Canine atopic dermatitis is a common allergic skin disease in dogs that causes chronic itching and recurrent skin inflammation. In clinical practice, veterinarians usually evaluate disease severity using owner-reported itch scores and clinician-based skin lesion scores. However, these assessments can vary and may not always reflect underlying immune activity. Blood-based biomarkers could provide additional objective information, but their usefulness in everyday clinical cases remains unclear. In this study, we measured five immune signaling molecules in the blood of 143 dogs, including healthy dogs and dogs with atopic dermatitis with or without systemic treatment. We then examined how these markers differed among groups and how they related to itch and skin lesion scores. We found that IL-31, a cytokine known to be involved in itch signaling, remained higher in dogs with atopic dermatitis than in healthy dogs, even during treatment, and was associated with owner-reported itching. In contrast, IFN-γ showed negative associations with both itch and lesion severity. These findings suggest that certain blood cytokines may provide useful complementary information when interpreting clinical signs and may indicate ongoing immune activity even if dogs appear clinically improved.

## 1. Introduction

Canine atopic dermatitis (cAD) is one of the most common chronic inflammatory skin conditions in dogs and is characterized by severe pruritus and recurrent erythema. Its pathogenesis is considered multifactorial and involves genetic predisposition, impairment of the skin barrier, and complex hypersensitivity immune responses to environmental allergens [1,2].

At present, no single biomarker has been established as a gold standard for the definitive diagnosis of cAD. Therefore, in clinical practice, the diagnosis is generally made by excluding other causes of pruritus, such as infectious diseases and ectoparasitic infestations, and by applying clinical diagnostic criteria, including Favrot’s criteria [3,4]. However, this diagnostic approach may be influenced by variability in clinical presentation and differences in clinical interpretation. Accordingly, there is an ongoing need for biomarkers that can objectively assess and monitor disease activity in real-world clinical settings.

In clinical practice, the severity of cAD is commonly assessed using the pruritus visual analog scale (pVAS), reported by owners, and the Canine Atopic Dermatitis Extent and Severity Index (CADESI-04), evaluated by clinicians [5,6]. Nevertheless, it remains unclear whether these indices reflect not only local skin inflammation but also systemic immune and inflammatory changes measurable in blood, as previous studies have reported inconsistent findings [7,8,9]. Moreover, many previous studies have primarily investigated early-stage or untreated patients; therefore, limited information is available regarding serum immune biomarker patterns in heterogeneous clinical populations that include dogs receiving systemic therapy, as commonly encountered in practice [8,10,11]. Th2-related cytokines (IL-13 and IL-31), the Th1 cytokine IFN-γ, and regulatory cytokines associated with Treg responses (IL-10 and TGF-β1) have been proposed as important immune mediators involved in the pathophysiology of cAD [7,12,13]. However, how the concentrations of these mediators in treated patients change in parallel with clinical signs (pVAS, CADESI-04), or what relationships they show with these indices, has not yet been clearly established [8,9].

Accordingly, in this study, client-owned dogs were categorized into three groups: healthy dogs (Healthy), dogs with atopic dermatitis not receiving systemic therapy (Untreated AD), and dogs with atopic dermatitis receiving systemic therapy (Treated AD). We then compared serum concentrations of selected cytokines (IFN-γ, IL-10, IL-13, IL-31, and TGF-β1) among these groups. The study aimed to characterize systemic cytokine profiles in dogs with cAD and to evaluate whether these immunologic markers differ between healthy dogs and dogs with cAD according to treatment status. In addition, correlations between cytokine concentrations and clinical severity indices (pVAS and CADESI-04) were analyzed to explore their potential as complementary biomarkers in clinical practice.

These analyses were designed to improve understanding of the relationship between circulating cytokines and clinical severity in canine atopic dermatitis and to determine whether selected serum cytokines may provide objective information that complements clinical scoring systems used in routine veterinary practice.

## 2. Materials and Methods

### 2.1. Study Populations

A total of 143 dogs were included in the study, comprising a group of healthy dogs (*n* = 28) and dogs with cAD (*n* = 115). The control group consisted of clinically healthy dogs with no prior history of dermatologic diseases and no abnormalities on physical examination. cAD was diagnosed when dogs met at least five of the eight criteria proposed by Favrot et al. [4], and infectious causes of pruritus, including pyoderma, ectoparasitic infestations, and dermatophytosis, were excluded. All enrolled dogs received ectoparasite prevention and underwent a dietary elimination trial for at least 8 weeks.

Dogs were categorized into three groups according to treatment status at the time of blood sampling: Healthy (*n* = 28), dogs with no prior history of dermatologic diseases; Untreated AD (*n* = 27), dogs with cAD that had not received any systemic antipruritic or immunomodulatory therapy for at least 4 weeks prior to sampling; and Treated AD (*n* = 88), dogs with cAD receiving systemic therapy at the time of sampling (prednisolone, oclacitinib, lokivetmab, or cyclosporine). No other forms of cAD management, such as allergen-specific immunotherapy, topical corticosteroids, or antimicrobials, were included in this study. Dogs receiving systemic therapy were analyzed as a single treated group without stratification by medication type, reflecting treatment patterns in routine clinical practice rather than drug-specific effects. This study was designed as a cross-sectional observational study, and medication selection was determined based on clinical necessity. To minimize potential confounding effects, dogs that had received systemic immunomodulators or other antipruritic medications not included in the study protocol within 4 weeks prior to sampling were excluded.

### 2.2. Ethical Considerations

This study was approved by the Institutional Animal Care and Use Committee (approval no. VNG-25-002-1) and was conducted in client-owned dogs presented to a veterinary hospital in Daejeon, Republic of Korea, between April 2025 and November 2025. Written informed consent was obtained from all owners prior to participation in the study. To avoid causing unnecessary pain or stress to the animals, residual blood samples collected during routine diagnostic testing and clinical care were used. No additional blood sampling was performed solely for research purposes.

### 2.3. Sample Collection and Storage

Blood samples were collected from the jugular vein into serum separator tubes (SST) and allowed to clot at room temperature for 30 min prior to centrifugation. Samples were centrifuged at 3000 rpm for 15 min to obtain serum. The separated serum was aliquoted into cryovials and initially stored at −20 °C. Within 2 weeks, samples were transferred to −80 °C and stored at −80 °C until cytokine analysis. Freeze–thaw cycles were limited to a single thaw prior to analysis.

### 2.4. Assessment of Clinical Severity

Clinical severity was assessed using the owner-reported pruritus score (pVAS) and the clinician-assessed lesion severity index (CADESI-04) [9]. The pVAS was scored on a scale from 0 (no pruritus) to 10 (most severe pruritus) using the validated descriptive scale completed by the owners. In the analysis, we used the owner-reported pVAS score as a continuous variable without applying a predefined cutoff. CADESI-04 scores were determined by summing lesion severity scores (erythema, lichenification, and excoriation/alopecia) across 20 predefined body sites. Based on CADESI-04 scores, disease severity was categorized as remission (0–9), mild (10–34), moderate (35–59), and severe (≥60). Because the number of dogs in the moderate and severe categories was limited, these groups were combined for statistical analysis. Associations between clinical severity indices and cytokine concentrations were subsequently evaluated.

### 2.5. Measurement of Cytokines

Five cytokines (IFN-γ, IL-10, IL-13, IL-31, and TGF-β1) were measured in serum samples stored at −80 °C using an enzyme-linked immunosorbent assay (ELISA). Hemolyzed or lipemic serum samples were excluded from the analysis, and freeze–thaw cycles were limited to a single thaw prior to analysis. All samples were measured once without duplicate analysis, and intra-assay and inter-assay variabilities were not independently assessed.

The ELISA kits used were as follows: IFN-γ (CAIF00, R&D Systems, Minneapolis, MN, USA), IL-10 (MBS450823, MyBioSource, San Diego, CA, USA), IL-13 (CI0043, NeoBiolab, Cambridge, MA, USA), IL-31 (CI0041, NeoBiolab, Cambridge, MA, USA), and TGF-β1 (USEA124CA, USCN, Wuhan, China). All assays were performed according to the manufacturers’ instructions, consistent with standard ELISA methodologies described in previous studies evaluating serum cytokines in dogs [14]. IFN-γ, IL-10, and TGF-β1 were quantified using a sandwich ELISA, whereas IL-13 and IL-31 were quantified using a competitive ELISA. Absorbance was measured at 450 nm using a microplate reader, and cytokine concentrations were calculated from standard curves generated for each assay, and all samples were processed under consistent experimental conditions to minimize technical variability. The assay sensitivity or detection range for each cytokine was defined according to the manufacturers’ specifications.

### 2.6. Statistical Analysis

Data were summarized as mean ± standard deviation (SD) and median values. Comparisons of cytokine concentrations among the three groups (Healthy, Untreated AD, and Treated AD) were performed using the Kruskal–Wallis test. When significant differences were detected, post hoc pairwise comparisons were conducted using the Mann–Whitney U test with Bonferroni correction. For the three pairwise comparisons among the three groups, the adjusted significance level was set at *p* < 0.0167.

Comparisons of cytokine concentrations among groups classified by CADESI-04 (Healthy, remission [0–9], mild [10–34], and ≥35) were also conducted using the Kruskal–Wallis test. Because the number of dogs classified as moderate (35–59) and severe (≥60) was limited, these categories were combined into a single moderate-to-severe group (≥35) for statistical analysis. When significant differences were detected, post hoc analyses were performed using the Mann–Whitney U test with Bonferroni correction. For the six pairwise comparisons among the four groups, the adjusted significance level was set at *p* < 0.0083.

Correlations between cytokine concentrations and the pVAS or CADESI-04 were assessed using Spearman’s rank correlation coefficient. Statistical significance was set at *p* < 0.05. All statistical analyses were performed using SPSS Statistics version 22.0 (IBM Corp., Armonk, NY, USA).

## 3. Results

### 3.1. General Characteristics of the Study Population

A total of 143 dogs were included and categorized into three groups according to treatment status at the time of blood collection: Healthy (*n* = 28), Untreated AD (*n* = 27), and Treated AD (*n* = 88).

The mean age was 6.93 ± 3.34 years in Healthy dogs, 8.44 ± 3.79 years in Untreated AD dogs, and 7.58 ± 3.42 years in Treated AD dogs. Sex distribution differed among groups. The Healthy group included 7 intact males, 3 intact females, 10 neutered males, and 8 spayed females. The Untreated AD group consisted of 20 neutered males and 7 spayed females. The Treated AD group included 3 intact males, 11 intact females, 47 neutered males, and 27 spayed females (Table 1).

Among dogs with AD (*n* = 115), the most common breeds were Maltese (30 dogs, 26.09%), Poodle (24 dogs, 20.87%), Bichon Frise (20 dogs, 17.39%), Pomeranian (10 dogs, 8.70%), and Shih Tzu (8 dogs, 6.96%) (Table 2).

### 3.2. Comparison of Serum Cytokine Concentrations Among Groups

Serum cytokine concentrations were compared among the Healthy, Untreated AD, and Treated AD groups. Significant differences among groups were observed for IFN-γ (*p* < 0.001), IL-13 (*p* = 0.001), and IL-31 (*p* = 0.004), whereas IL-10 (*p* = 0.213) and TGF-β1 (*p* = 0.224) did not differ significantly.

Serum IFN-γ concentrations were significantly lower in the Treated AD group (194.56 ± 26.95) than in the Healthy (204.78 ± 17.23) and Untreated AD (201.76 ± 15.34) groups (*p* < 0.001).

Serum IL-13 concentrations were significantly lower in the Treated AD group (711.09 ± 472.26) than in the Healthy (1036.46 ± 557.43) and Untreated AD (1002.21 ± 444.57) groups (*p* = 0.001).

**Figure 1:**
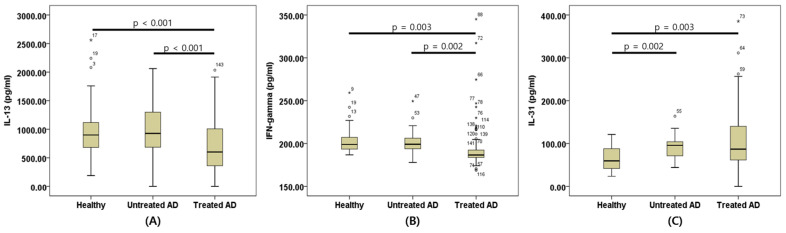
Box-plot comparison of serum cytokine concentrations among Healthy, Untreated AD, and Treated AD dogs. Box-plots show serum IL-13 (**A**), IFN-γ (**B**), and IL-31 (**C**) concentrations in the three groups. The horizontal line within each box represents the median, boxes indicate the interquartile range, and circles and asterisks denote outliers. AD: Atopic dermatitis; IL-13, Interleukin-13; IFN-γ: Interferon-gamma; IL-31: Interleukin-31.

Serum IL-31 concentrations were significantly higher in the Untreated AD (91.02 ± 27.15) and Treated AD (102.94 ± 67.95) groups than in the Healthy group (64.37 ± 28.97) (*p* = 0.004; Table 3, Figure 1).

### 3.3. Correlations Between pVAS and Serum Cytokine Concentrations

Correlation analyses between the pVAS and serum cytokine concentrations were performed in all 143 dogs. In Spearman’s correlation analysis, the pVAS showed a significant negative correlation with IFN-γ (r = −0.239, *p* = 0.004) and a significant positive correlation with IL-31 (r = 0.173, *p* = 0.039).

**Figure 2:**
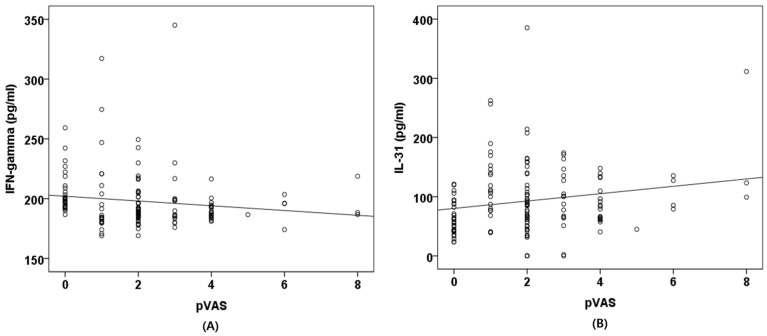
Correlation between pVAS and serum cytokine concentrations. Scatter plots illustrate the relationships between pVAS scores and serum concentrations of IFN-γ (**A**) and IL-31 (**B**). Each point represents an individual dog. pVAS: Pruritus visual analog scale; IFN-γ: Interferon-gamma; IL-31: Interleukin-31.

In contrast, IL-10 (r = −0.065, *p* = 0.442), IL-13 (r = −0.057, *p* = 0.500), and TGF-β1 (r = 0.017, *p* = 0.844) were not significantly correlated with the pVAS (Figure 2).

### 3.4. Correlations Between CADESI-04 and Serum Cytokine Concentrations

Correlation analyses between the CADESI-04 scores and serum cytokine concentrations were performed in all 143 dogs. In Spearman’s correlation analysis, the CADESI-04 scores showed a significant negative correlation with IFN-γ (r = −0.252, *p* = 0.002).

**Figure 3:**
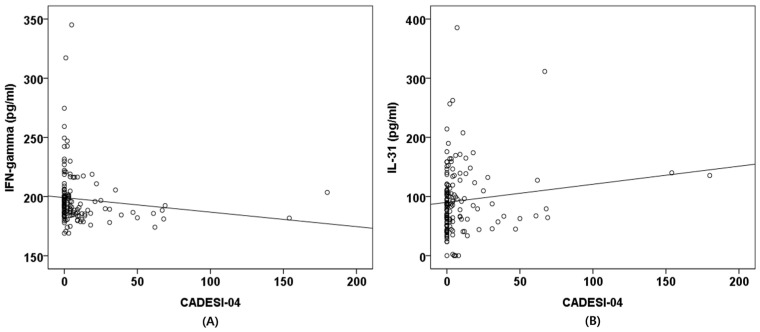
Correlation between CADESI-04 scores and serum cytokine concentrations. Scatter plots illustrate the relationships between CADESI-04 scores and serum concentrations of IFN-γ (**A**) and IL-31 (**B**). Each point represents an individual dog. CADESI-04: Canine atopic dermatitis extent and severity index-04; IFN- γ: Interferon-gamma; IL-31: Interleukin-31.

In contrast, correlations between CADESI-04 scores and IL-10 (r = −0.066, *p* = 0.433), IL-13 (r = −0.096, *p* = 0.253), IL-31 (r = 0.128, *p* = 0.128), and TGF-β1 (r = −0.022, *p* = 0.799) were not significant (Figure 3).

### 3.5. Comparison of Serum Cytokine Concentrations Among CADESI-04 Severity Groups

Based on the CADESI-04 scores, dogs were classified into four groups: Healthy (*n* = 28), remission (*n* = 86), mild (*n* = 18), and moderate to severe (*n* = 11). Serum cytokine concentrations were compared among these groups.

Serum IFN-γ concentrations differed significantly among the four groups (*p* < 0.001). In post hoc analyses, the Healthy group showed significantly higher IFN-γ concentrations than the remission (*p* = 0.001), mild (*p* < 0.001), and moderate-to-severe (*p* = 0.001) groups, and these differences remained significant after Bonferroni correction (*p* < 0.0083).

Serum IL-31 concentrations also differed significantly among groups (*p* = 0.01). In post hoc analyses, the Healthy group showed lower IL-31 concentrations than the remission (*p* = 0.001), mild (*p* = 0.023), and moderate-to-severe (*p* = 0.042) groups. After Bonferroni correction (*p* < 0.0083), only the difference between the Healthy and remission groups remained significant.

**Figure 4:**
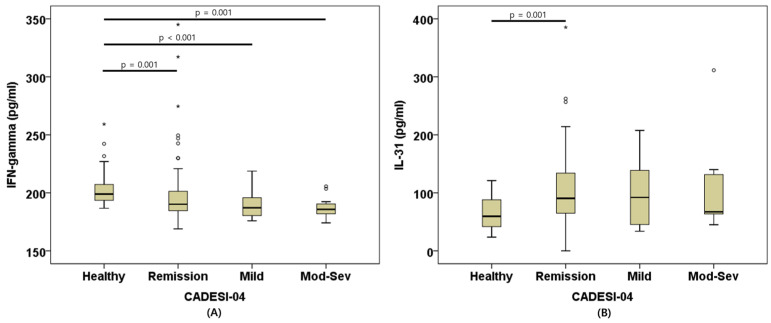
Comparison of serum cytokine concentrations according to CADESI-04 severity groups. Box plots show serum IFN-γ (**A**) and IL-31 (**B**) concentrations among Healthy, remission, mild, and moderate-to-severe groups. The horizontal line represents the median, and the boxes indicate the interquartile range. Circles and asterisks denote outliers. IFN-γ, Interferon-gamma; IL-31, Interleukin-31; CADESI-04, Canine atopic dermatitis extent and severity in-dex-04.

In contrast, IL-10, IL-13, and TGF-β1 did not show significant differences among the severity groups (Figure 4).

## 4. Discussion

In this study, we evaluated the serum concentrations of five cytokines (IFN-γ, IL-10, IL-13, IL-31, and TGF-β1) in client-owned dogs with canine atopic dermatitis and investigated their associations with clinical severity indices (pVAS and CADESI-04). The principal findings were that serum IL-31 concentrations were elevated in dogs with atopic dermatitis compared with healthy controls and were positively correlated with the pVAS, whereas IFN-γ concentrations showed negative correlations with both the pVAS and CADESI-04. In contrast, IL-10, IL-13, and TGF-β1 did not demonstrate consistent associations with disease presence or clinical severity.

IL-31 is widely recognized as a key pruritogenic cytokine in canine atopic dermatitis [12,15]. In the present study, serum IL-31 concentrations were significantly higher in dogs with atopic dermatitis than in healthy controls, and a positive correlation was observed between IL-31 and the pVAS. These findings are consistent with previous studies suggesting that IL-31 plays a central role in the pruritus pathway of canine atopic dermatitis and that circulating IL-31 may reflect pruritus-related immune signaling [9,12,15]. Interestingly, serum IL-31 remained elevated compared with that of healthy controls even in dogs classified as being in remission based on the CADESI-04. This observation suggests that immunologic activity related to pruritus may persist even when clinical signs appear well controlled. Such dissociation between clinical improvement and underlying immune signaling may reflect subclinical inflammation or residual cytokine activity that is not fully captured by clinical scoring systems. This observation may also have practical implications, suggesting that cytokine-based biomarkers could provide complementary information beyond clinical scoring systems when evaluating treatment responses. These findings should be interpreted in the context of heterogeneous treatment mechanisms, as different systemic therapies may exert distinct effects on cytokine regulation. It is also conceivable that clinical improvement may involve modulation of the IL-31 receptor expression or downstream signaling pathways, such as the JAK/STAT pathway, although these mechanisms were not directly evaluated in this study.

In contrast to its relationship with the pVAS, IL-31 did not show a statistically significant correlation with the CADESI-04 in this study. This finding may be explained by differences in the biological processes represented by these indices. While the pVAS primarily reflects pruritus intensity perceived by the owner, the CADESI-04 represents the cumulative burden of visible skin lesions, including erythema, lichenification, excoriations, and alopecia. Lesional changes often resolve more slowly than pruritus during treatment, which may weaken the statistical association between IL-31 and the CADESI-04. Similar discrepancies between IL-31 and lesion-based indices have been reported previously [9,16,17]. These findings support the interpretation that IL-31 may be more closely associated with pruritus signaling rather than overall lesion burden.

IFN-γ is a representative Th1-associated cytokine involved in cellular immune responses. In canine atopic dermatitis, immune responses are generally considered to be dominated by Th2-related pathways, particularly during early phases of the disease, whereas Th1 responses may become more prominent as the disease progresses into a chronic stage [18,19]. However, evidence for increased IFN-γ has largely been derived from analyses of local cutaneous lesions rather than the systemic circulation [20,21]. Previous studies evaluating serum IFN-γ concentrations in dogs with atopic dermatitis have reported inconsistent findings, including no significant differences compared with healthy controls, concentrations below detection limits, or decreased levels in affected dogs [10,11,22].

In the present study, serum IFN-γ concentrations showed negative correlations with both the pVAS and the CADESI-04, indicating a weak negative relationship between systemic IFN-γ levels and clinical severity indices in this study. Interestingly, IFN-γ concentrations were significantly lower in systemically treated dogs than in healthy controls or untreated dogs. Several medications included in the Treated group, such as prednisolone, cyclosporine, and oclacitinib, are known to suppress T-cell-mediated immune responses. Therefore, the observed reduction in IFN-γ may partially reflect treatment effects rather than disease activity alone [23,24,25]. At the same time, the cross-sectional design of this study limits the ability to distinguish treatment-induced changes from baseline immunologic differences between groups. Consequently, the reduced IFN-γ concentrations observed in treated dogs should be interpreted cautiously and may reflect a combination of pharmacologic suppression and underlying disease characteristics. Given that the treated group included drugs with distinct immunomodulatory mechanisms, these findings should be interpreted cautiously, as the observed cytokine changes may reflect heterogeneous treatment effects rather than a uniform biological response. Given that higher CADESI-04 scores may reflect chronic dermatologic changes such as lichenification, the observed relationship between IFN-γ and clinical indices may also warrant consideration in the context of disease chronicity.

Serum IL-13 did not differ significantly between untreated dogs with atopic dermatitis and healthy controls, although concentrations were lower in the treated group. IL-13 is a Th2-associated cytokine involved in allergic inflammation and skin barrier dysfunction. IL-13 has also been associated with the impairment of epidermal barrier function through the suppression of proteins such as filaggrin, which may contribute to chronic lesion remodeling in atopic dermatitis. In human studies, increased IL-13 expression has been associated with reduced filaggrin expression, barrier dysfunction, and fibrosis [26,27]. Previous experimental studies have suggested that IL-13 expression may increase in lesional skin following allergen exposure, and may increase more slowly than IL-31 following allergen exposure [28]. In the present study, however, the untreated group mainly consisted of dogs at a stage where systemic treatment was not yet required, rather than dogs with severe untreated disease, and most were classified as being in remission or mild based on the CADESI-04. This may explain why serum IL-13 did not differ significantly from that in healthy dogs, as substantial barrier dysfunction or chronic lesion remodeling may not yet have been sufficiently established in these dogs. Therefore, IL-13 may be more closely linked to barrier dysfunction and chronic lesion development than to pruritus itself. In contrast, the lower IL-13 concentrations observed in the treated group may reflect treatment-related immunomodulatory effects.

Serum IL-10 and TGF-β1, both regulatory cytokines associated with immune modulation and regulatory T-cell activity, did not show consistent associations with disease presence or clinical severity in this study. Previous investigations in both canine and human atopic dermatitis have also reported inconsistent findings regarding circulating regulatory cytokines [8,21,29,30]. One possible explanation is that TGF-β1 is not exclusively derived from regulatory T cells but can be produced by multiple cell types, which may obscure its relationship with disease-specific immune activity in the systemic circulation [31,32]. Additionally, serum concentrations of these cytokines are often low and may show substantial inter-individual variability, which can limit their usefulness as systemic biomarkers in cross-sectional analyses. For this reason, these cytokines may be less suitable as standalone systemic biomarkers for evaluating disease activity in canine atopic dermatitis.

Several limitations of this study should be acknowledged. First, this was a cross-sectional observational study based on client-owned dogs presented to a single veterinary hospital, and causal relationships between cytokine concentrations and disease activity cannot be established. Second, treatment allocation was not randomized and was determined according to clinical necessity; therefore, differences in cytokine concentrations may reflect both treatment effects and baseline clinical characteristics. Third, the Untreated group contained a large proportion of dogs with mild disease, which may have influenced the detection of cytokine differences across severity categories. Finally, the Treated group included multiple systemic therapies with different mechanisms of action. Because these treatments were analyzed as a single group, the observed cytokine patterns should be interpreted cautiously, as they may reflect heterogeneous treatment effects rather than a uniform treatment-related response. In addition, cytokine measurements were performed without duplicate analysis and assay variability was not independently assessed, which may limit the precision of the measurements. Future studies should therefore evaluate drug-specific cytokine profiles to better clarify treatment-related immunologic changes.

Despite these limitations, the present study provides insight into systemic cytokine patterns in dogs with naturally occurring atopic dermatitis in a clinical setting. In particular, the consistent association between serum IL-31 and pruritus scores supports the potential relevance of IL-31 as a candidate biomarker reflecting pruritus-related immune signaling. In addition, the negative relationship between IFN-γ and clinical severity suggests that the systemic immune balance may shift with disease activity and treatment. These findings suggest that selected serum cytokines may be associated with clinical signs, although their usefulness as biomarkers should be interpreted cautiously. Future longitudinal studies following individual dogs over time will be necessary to clarify the temporal relationship between changes in clinical signs and circulating cytokine profiles.

## 5. Conclusions

In this study, we compared serum cytokine concentrations among healthy dogs, untreated dogs with atopic dermatitis, and systemically treated dogs, and evaluated their relationships with the pVAS and the CADESI-04. Among the cytokines analyzed, IFN-γ, IL-13, and IL-31 showed significant differences among groups. IFN-γ and IL-13 concentrations were lower in treated dogs than in healthy dogs, whereas IL-31 concentrations were higher in dogs with atopic dermatitis than in healthy dogs, regardless of treatment status. In correlation analyses, IL-31 showed a positive association with the pVAS but not with the CADESI-04, suggesting that IL-31 may be more closely related to pruritus signaling than to overall lesion severity. In contrast, IFN-γ showed negative correlations with both the pVAS and the CADESI-04, indicating a potential relationship between systemic immune balance and clinical disease activity. IL-10 and TGF-β1 did not show consistent associations with disease presence or clinical severity, suggesting limited utility as serum biomarkers under the conditions of this study.

Overall, these findings indicate that serum cytokine profiles and clinical indices do not necessarily change in parallel in cross-sectional analyses. In particular, the association between IL-31 and pruritus suggests that serum IL-31 may provide useful context for interpreting pruritus-related immune signaling that can persist even in clinically treated dogs, although these findings should be interpreted with caution given the heterogeneity of systemic treatment mechanisms.

## Figures and Tables

**Table 1:** Signalment of dogs in this study.

Characteristics		Healthy	Untreated AD	Treated AD
Number	N	28	27	88
	%	20	19	61
Age	Mean	6.93	8.44	7.58
	SD	3.34	3.79	3.42
Sex	Intact Male	7	0	3
	Intact Female	3	0	11
	Castrated male	10	20	47
	Spayed female	8	7	27

N: number; AD: Atopic dermatitis; SD: Standard deviation.

**Table 2:** Distribution of atopic dermatitis dogs.

Characteristics		N (%)	Untreated AD	Treated AD
Breed	Maltese	30 (26.09)	5	25
	Poodle	24 (20.87)	6	18
	Bichon Frise	20 (17.39)	6	14
	Pomeranian	10 (8.70)	3	7
	Shih Tzu	8 (6.96)	1	7
	Dachshund	4 (3.48)		4
	Coton De Tulear	4 (3.48)	1	3
	Golden Retriever	3 (2.61)		3
	Yorkshire Terrier	2 (1.74)		2
	Labrador Retriever	1 (0.87)		1
	French Bulldog	1 (0.87)	1	0
	Cocker Spaniel	1 (0.87)	1	0
	Cavalier King Charles Spaniel	1 (0.87)	1	0
	American Bully	1 (0.87)		1
	Jack Russell Terrier	1 (0.87)		1
	Mix	4 (3.48)	2	2

N: number; AD: Atopic dermatitis.

**Table 3:** Comparison of serum cytokine concentrations among Healthy, Untreated AD, and Treated AD groups.

Variable	Healthy	Untreated AD	Treated AD	*p*
	Mean (SD)	Mean (SD)	Mean (SD)	
IFN-γ	204.78 (17.23)	201.76 (15.34)	194.56 (26.95)	<0.001 ***
IL-10	99.32 (173.20)	49.08 (4.93)	83.74 (185.82)	0.213
IL-13	1036.46 (557.43)	1002.21 (444.57)	711.09 (472.26)	0.001 **
IL-31	64.37 (28.97)	91.02 (27.15)	102.94 (67.95)	0.004 **
TGF-β1	34.34 (5.41)	35.78 (7.47)	35.62 (5.86)	0.224

AD: Atopic dermatitis; SD: standard deviation; IFN-γ: interferon-gamma; IL-10: interleukin-10; IL-13: interleukin-13; IL-31: interleukin-31; TGF-β1: transforming growth factor-beta1; Kruskal–Wallis test. ** *p* < 0.01, *** *p* < 0.001.

## Data Availability

The data presented in this study are available on request from the corresponding authors for reasonable reasons.
